# Which One Is More Prominent in Recurrent Hydatidiform Mole, Ovum or Sperm?

**DOI:** 10.22074/ijfs.2020.6017

**Published:** 2020-07-15

**Authors:** Maryam Hafezi, Zahra Chekini, Mohammadreza Zamanian

**Affiliations:** 1 Department of Endocrinology and Female Infertility, Reproductive Biomedicine Research Center, Royan Institute for Reproductive Biomedicine, ACECR, Tehran, Iran.; 2 Department of Genetics, Reproductive Biomedicine Research Center , Royan Institute for Reproductive Biomedicine, ACECR, Tehran , Iran.

**Keywords:** Hydatidiform Mole, Intracytoplasmic Sperm Injections, Ovum Donation, Preimplantation Genetic Diagnosis

## Abstract

Recurrent hydatidiform mole is defined as episodes of two molar pregnancies in a female. Often, complete moles only derive androgenic nuclear genome. We described two cases with repeated molar pregnancies attempted to prevent future episodes by performing intracytoplasmic sperm injection (ICSI) and preimplantation genetic diagnosis (PGD) to assess genetic disorders. The first patient had previously six complete molar pregnancies and advised to carry out ICSI with ovum donation to achieve a normal pregnancy. The second case had previously five molar pregnancies and no XY embryos from the ICSI/PGD process. We had to (at the insistence of the patient) transfer XX embryos in this patient which resulted in a complete hydatidiform mole (CHM). Hence, available data based on our patients and previous studies demonstrated that oocyte might play a critical role in the pathophysiology of recurrent hydatidiform mole, while it has not been often considered.

## Introduction

Complete hydatidiform mole (CHM) is characterized by diffuse chorionic villi hyperplasia and generalized hydatidiform villous swelling (1). Recurrent hydatidiform mole is an extremely rare occurrence. Rate of that is more than 23% after two molar pregnancies in the same woman (2, 3). Recurrent CHM is related to a higher malignancy risk (4). Early abortions in approximately 10-20% after one hydatidiform mole show the genetic origin of molar pregnancies in some of these patients (1, 5). Furthermore, there is 44-66% chance of live births at future pregnancies (6).

Molar pregnancy has a multifactorial etiology related to several environmental and genetic factors (7). Complete moles usually have their nuclear genome from the paternal (androgenesis). In such cases, chromosomal material from the ovum is lost or becomes inactive, whereas the mitochondrial DNA has maternal origin (1, 8).

Preliminary investigation for prevention of CHM was based on the morphological manifestation of embryos during in vitro fertilization (IVF) (9). However, according to genetic composition and pathogenesis of molar pregnancies, intracytoplasmic sperm injection (ICSI) and preimplantation genetic diagnosis (PGD) with fluorescent in situ hybridization (FISH) provide a diploid 46, XY complement which is appropriate for prevention of an additional event in patients with repeated molar pregnancies (6). Defective oocytes can be a predisposing factor as the main cause of abnormal fertilization thus an oocyte or embryo donation is considered for achieved normal pregnancy (10, 11).

In the present study, we described two cases of recurrent molar pregnancy which were advised to have ICSI/PGD to prevent repeated CHM. This led to their molar pregnancies. Oocyte donation in the current cases resulted in normal pregnancies and live births.

## Case Report

### Case 1

A 30-years-old woman presented with six molar pregnancies and five suction curettages in the last nine years. All pregnancies had complete molar pathology. The patient underwent subfertility treatment for nine years and had conceived by ovarian stimulation with clomiphene in all pregnancies, which led to histopathological diagnoses of hydatidiform mole. She had regular menstrual cycles and a body mass index (BMI) of 29kg/m2. The patient had no history of blood transfusions and no addictions. Her blood group was B positive and she had normal thyroid hormone profile. Immunological assessments such as anti-phospholipid (IgG and IgM) and anti-cardiolipin antibodies, anti-ds-DNA, anti-nuclear antibody (ANA), lupus anti-coagulant and CHso were performed due to her recurrent abortion history. She was negative for any autoimmune disorders. Pelvic examination showed remarkable deformation and scarring in the cervix, resulted from tenaculum lesions. Her husband was 37 years old with the following semen indices: concentration of 120×10^6^/ ml with normal motility (40%) and normal morphology (21%) according to Kruger's criteria evaluation. Both of the patient and her partner had normal karyotypes. It was not consanguine marriage.

Genetic counseling recommendations included ICSI/ PGS. The first ovarian stimulation was achieved by the use of oral contraceptive pill (OCP) long GnRHa. Combined low-dose (LD) contraceptive pills (Abureyhan Pharmaceutical Company, Iran) starting on day 2 of the menstrual cycle then buserelin (Suprefact; Hoechst, Denmark) was initiated from day 17^th^ of the cycle. After pituitary down-regulation was achieved, Gonal-F was subcutaneously injected for nine days at a dose of 150 IU/day (Serono, Switzerland). Ovarian response was monitored by vaginal ultrasound when two follicles had diameters of more than 17 mm and ovulation was induced by administration of 10000 IU of human chorionic gonadotropin (hCG; Pregnyl, Germany). A total of 12 oocytes were retrieved 36 hours post-hCG administration. The number of metaphase II (MII) oocytes was evaluated and the patient underwent insemination procedures. Ten embryos were obtained at the cleavage stage. An advanced cleavage status of an 8-cell embryo at 72 hours after insemination resulted in four embryos with two-pronuclear (PN), three embryos with 1.PN, one embryo without PN and 2 embryos with three-PN and good and fair grading. All embryos proceeded to the cleaving stage (day 3 after retrieval) and were biopsied for single-cell PGS by FISH. FISH probes were specific for chromosomes 13, 18 and 21 to determine aneuploidy. Two technologists were evaluated and construed the signals of FISH probes. We observed micronuclei and extra nucleus in all embryos that were not transferred due to the presence of multi-micronuclei embryos (Table 1)

After three years and based on the patient's history, the couple was advised to have an oocyte donation and ICSI procedure. Ovarian stimulation of the donors was performed using a standard oral contraceptive pills (OCP) long GnRHa stimulation protocol and pituitary-ovarian suppression by buserelin. There were 21 out of 26 oocytes at the MII stage.

ICSI procedure was routinely performed to prevent dispermia. We obtained 20 2.PN good grade embryos from insemination. Three cleaving excellent embryos were transferred 2 days after ICSI procedure without any positive result. Other frozen embryos were transferred three times in three years. The second cycle resulted in pregnancy and live birth, by transferring three good embryos.

One year after delivery of the first live birth, an endometrial adhesion was detected by ultrasonography examination. A hysteroscopy was subsequently performed. During the second donor cycle, four oocytes were retrieved according to the previous donor protocol and three excellent stage 2.PN embryos were transferred. Pregnancy was confirmed by a persistent rise in serum β-hCG levels on the 14th day after embryo transfer. Ultrasonography at six weeks gestation was remarkable for a singleton pregnancy with a positive fetal heart rate. She eventually had vaginal delivery of a healthy infant. The recruited patient gave her consent to participate.

**Table 1:** Details of biopsy and spreading of embryos from the second case and the respective results of FISH analysis


Embryo Grade	No. Cells biopsied	Nucleus	Fragmentation	PGD FISH results	Whole embryo FISH results	Embryos status in transfer day

B	3/8	3.PN	+	?#	-	5B
B	1/8	2.PN	+	[13][18][13][18][21×3]	-	8B
B-C	1/10	1.PN	+	[13×2][18][21×2]	-	Compact
B	1/10	1.PN	+	[13][18×2][21]	^-^	Compact
C	1/8	2.PN	+	[13][18×3][21×2] [13][21]	-	7C
C	1/6	NO*	+	[13][18][21]	-	5C
C	1/4	2.PN	+	[13×3][18×3][21×4]	-	6C
C	1/6	2.PN	+	No signal detected	-	8BC
C	1/4	1.PN	+	[13×2][18×2][21×2]	-	3C
C-D	1/4	3.PN	+	[13][18][21]×2[13][18]	-	4CD


*; No nucleus was observed, # ; Not available, PGD; Genetic diagnosis, FISH; Fluorescent in situ hybridization,? Unknown, and PN; pronucleus.

### Case 2

A 24-years-old woman had complaints of consecutive CHM verified by histopathology assessment of the evacuated uterine contents. Her medical history included five previous molar pregnancies occurred over 8 years following spontaneous conceptions that did not continue beyond the first trimester. The latest molar pregnancy occurred one year before admission. The patient married 12 years ago and she had no infertility history. Her BMI was 36 kg/ m2 and the blood group was O positive. She had regular menstrual cycles and normal thyroid profile. All infection tests were also normal. Hormonal assessment on day 3 of the menstrual cycle indicated an FSH level of 7.5 mIU/ ml and LH level of 4.93 mIU/ml. Abdominal ultrasonography revealed a 13 mm diameter isoechoic myometrial fibroid without any pressure effect on the endometrium.

**Table 2:** Details of biopsy and spreading of embryos as well as the results of FISH analysis


Embryo Grade	No. Cells biopsied	Nucleus	Fragmentation	PGD FISH results	Whole embryo FISH results	Embryos status in transfer day

B	1/8	2.PN	-	XX[18]×2	Transferred	Compact
B	1/4	2.PN	-	XX[18]×2	Transferred	Compact
C	1/8	2.PN	-	XY[18]?^*^	-	Compact
C	1/4	2.PN	-	Y ?	-	5C
B	1/8	1.PN	+	Y[18]	-	Compact
B	2/8	1.PN	+	Y[18]	-	Compact
C	1/6	-	-	?	-	Compact
C	2/8	3.PN	+	X[18] X ?	-	Compact


*; Not available, #; Binucleated cell with a normal-size nucleus and additional smaller nucleus, PGD; Genetic diagnosis, FISH; Fluorescent in situ hybridization (FISH results summarize both nuclei), ?:Unknown, and PN; pronucleus.

Semen analysis showed a concentration of 70×106/ml according to Kruger criteria, along with 30% motility and 14% morphology. The patient’s husband was a smoker and allergic to plastic supplies due to his employment at the plastics production plant. The couple had normal karyotypes with no gross abnormalities. They had no consanguine marriage.

A nutritional counselor advised the patient to lose weight. After extensive counseling, the couple underwent ICSI-PGS, which resulted in 46, XY embryo transfers to prevent sperm chromosome duplication. After confirmation of satisfactory down-regulation with subcutaneous buserelin, she underwent ovarian stimulation using a standard long protocol induced by daily Gonal-F (150 IU; Serono, Switzerland) administration. When two dominant follicles reached greater than 18mm in diameter, she received hCG (Ovitrelle; Serono, Switzerland). There were 11 mature MII oocytes and one immature oocyte transvaginally retrieved 36 hours after hCG injection. There were 2.PN confirmed in 4 embryos and one embryo with 3.PN observed 18 hours after insemination. The remaining 4 embryos showed no evidence of fertilization (1.PN). The embryos developed to the eight-cell stage 72 hours after insemination and a single-cell PGS was performed. No evidence of blastomere fragmentation or irregularity was observed. FISH probes were specific for the 18, X, Y chromosomes to determine the ploidy and sex selection. In the first FISH round, two XY embryos were detected; however, the second analysis showed 45 Y and 23 XX embryos. Because the insistence of the patient, two XX embryos were transferred, because the 46, XY embryo was not acquired (Table 2).

Two weeks after transfer, the patient had a positive serum β-hCG test. Ultrasonography after 6 weeks revealed one gestational sac and regions of low echogenicity that became multicystic and hydropic chorionic villi suggestive of a CHM. Despite the use of ICSI/PGS procedure, histologic examination after suction evacuation confirmed diagnosis of CHM for the sixth time. Two recruited patients gave their consent to participate. Informed consent form was obtained and completed by participant.

## Discussion

It appears to be a crucial relationship within CHM, immature ovum and delayed fertilization. Another possibility is altered integrity of the zona pellucida prone to entering double spermatozoa (12). CHM is predominantly 46,XX due to androgenic duplication, whereas only rare cases present 46,XX or 46,XY conditions as a result of dispermia (6, 10). The 46,YY constitution has yet to be described, most likely because such embryos will not be developed beyond a few cells (13).

IVF is predisposed to multi-sperm fertilization as a mechanism, which most likely causes the formation of hydatidiform moles (12). ICSI can prevent polyploidy by assurance arrival of a single spermatozoa to oocyte, resulting in more reduced triploidy; however, probability of molar pregnancy still remains (14). Avoidance of triploidy and ensuring delivery of haploid spermatozoa by ICSI might be of the benefits for patients who have a history of the recurrent trophoblastic disorder (12). However, during ICSI cycles, morphological assessment of the embryo before transfer cannot prevent CHM (14). An alternative genetic approach to prevent recurrent CHM is ICSI/PGD via FISH technique that complies with prior knowledge of the pathogenesis of hydatidiform mole. ICSI is likely to result in diploid and monospermic fertilization, as well as PGD for male sex selection embryos that confirm diploidy and the chromosomal contribution of both parents during fertilization (6).

In both cases, the couples were advised by genetic counselors to undergo ICSI/PGS. In the first case, no embryo was transferred because all embryos had multimicronuclei. In the second case, there was no XY embryo resulted from the ICSI/PGS process, presenting the restricted problem of this technique. Because the insistence of the patient, we had to transfer XX embryos with the appearance of normal nuclei, which resulted in a CHM by triploidy of origin. Therefore, the ICSI/PGS process was not effective in preventing molar pregnancy.

Recent advances have shown androgenesis in at least 80% of CHM, however, one of the remaining pathophysiologies of cases may be diploid biparental (15). ICSI/ PGD is an appropriate method for androgenetic avoidance and triploid dispermic in origin CHM due to its pathogenesis that occurs at the time of fertilization. However, this approach does not completely prevent a recurrent diploid biparental hydatidiform mole (10).

Biparental diploids have one maternal set of chromosomes and one set of the chromosome complement from father (8, 10). Although maternal defect is more involved, it seems that both of the partners are involved in molar pregnancy (10). Considering the biparental origin of CHM and the implicated disturbance in oocyte meiosis (10, 11), donor oocyte IVF/ICSI might be a therapeutic modality to prevent recurrent CHM and achieve a normal pregnancy.

In the current study, the first patient was advised to conceive via ICSI with ovum donation. This case had two normal pregnancies and a healthy child after a two embryo transfer cycle from donated oocytes. Nevertheless, the patient who underwent ICSI/PGS with her oocytes did not obtain a normal embryo nucleus, suggesting a critical role of the ovum in the pathophysiology of molar pregnancy.

As mentioned above, maternal mitochondrial DNA of the entire oocyte genome contributes only to the fertilization in CHM (8). Altered expression of mitochondrial genes has been associated with gestational trophoblastic disease (GTD) (16). The involvement of multiple somatic mtDNA mutations in GTD have been proposed in the pathogenesis of CHM and tumor development (17).

Pan et al. have described maternally derived mtDNA in CHM based on the polymorphic D-loop region. However, since mtDNA is highly polymorphic and heteroplasmic, limited reports have described the mtDNA-transmission pattern in hydatidiform moles (18). Therefore, most likely in the current cases, maternal mtDNA was involved in the pathophysiological events of CHM.

One important limitation of our study is the rare occurrence of GTD and insufficient achievement cases. However, further studies with larger sample size would be needed to understand the designation of mtDNA and maternal genetics predisposing to GTD.

## Conclusion

With respect to the established ICSI/PGS strategy for prevention of recurrent molar pregnancy, this technique has restrictions of sperm duplication, defective oocytes and avoidance from XX embryos. It seems that ovum donation is better treatment option to achieve normal pregnancy in such cases. Available data based on our patients and previous studies indicate that oocytes might have a critical role in pathophysiology of recurrent HM, while it has not been considered in most of the studies.
